# Justifiable Scope of Medical Journal Advertising

**Published:** 1915-08

**Authors:** G. Henri Bogart

**Affiliations:** Paris, Illinois


### Justifiable Scope of Medical Journal Advertising.

BY DR. G. HENRI BOGART, PARIS, ILLINOIS.

To begin with the foundation, a good magazine cannot be published with due regard to material and typographical excellence, at the prevailing subscription rates, except for the income from advertisements. The advertisement in a medical journal should be something that will enable the reader to know of some commodity or service that appeals to him, and it should be of a quality that the publisher can guarantee. It must tell the doctor something that he should want to know; it should fill a want, it should not be allowed space unless it does convey a welcome message. Hence, we find the old-time ideal that only medical or surgical supplies, sanitariums or retreats should find place in the journal, and these only when accepted as "regular," is on the run. The medical journal owes a duty to its readers. If there be products or opportunities of special interest to a profession, it is the duty of the publisher to bring the buyer and the seller together for their mutual benefit, for the publication which fills its place in the sun must be guided steadfastly by the ideal of service. For instance, a journal circulating in territory in which almost every doctor drives a car, is carrying a dozen pages of ads of automobile accessories. That is legitimate material for advertising, for the doctor learns of little things that will make his car more available, more comfortable or safer, and at the same time the advertiser is getting a square deal, since his message reaches a class almost universally interested in that subject.

The doctor lives a little better than the common laborer; he likes to have something that adds to the comfort of himself and family; he likes to have and should have a living worthy his position in the social scale of living, and any clean, uplifting element of better living is appropriate and desirable in the advertising pages of a medical journal.

Tropical fruits, food products, for instance, or musical instruments, good books and magazines are perfectly legitimate advertising in a medical journal. There is no medium that will give so large a percentage of probable customers to the advertiser as the medical journal, for the professional class that reads its pages is a purchasing class.

Then, too, the doctor's purchases are seen and commented on by a larger proportion of neighbors than any other, particularly so in the case of the country physician, and his recommendation gives the advertisement greater publicity than that of any other class of readers.

We must remember that this is the age of progress, that the old order changeth with kaleidoscopic rapidity, as in the so-called classified advertisement.

Only yesterday, as it were, the personal advertisement was found only in the columns of the daily newspapers, but within the past few months the great literary magazines have added departments of this style, the *Review of Reviews*, *Collier's*, and *Cosmopolitan* being examples.

It is really the duty of medical journals to carry a line of this advertisement. Suppose that Dr. A., in the north corner of the State has an office appliance which he wishes to sell. Dr. B., down in the south wants such an article, and the only possible means of their ever knowing of their mutual needs, is that medical journals shall act as the intermediary of communication. Dr. C. wants an assistant, but Dr. D., looking for a location across the State, has no means of knowing about it, unless the professional journals shall bear the tidings. There are an unbelievably large number of professional wants that can reach the profession only through the pages of the State journal.

There are a few, possibly half a dozen, medical journals in the country that have introduced this useful department into their advertising pages, and there is no department that is more eagerly sought by the average reader than this list of professional business opportunities.

For the advertiser, there is no more fertile field for high class advertisement of good stable articles of comfort or of minor luxury; on the other hand, the doctor can find no better or more reliable guide to the purchase of many commodities, provided the editor exercise the usual editorial vigilance in verifying the statements which he admits to his columns.

The medical folks do not expend all their incomes in the purchase of drugs and surgical instruments, much of their expenditure is for living, and any article which will appeal specially to the wants of this prominent and progressive class of American citizenship, is justifiably appropriate in a medical journal.

The best service to the profession that a medical journal can give is in supplying reliable information that will minister to the well being of the doctor and those dependent upon him.

The doctor is a citizen—and usually a mighty good sort of citizen—before he is a physician, and the aloofness that would segregate him from his fellows is pretty well broken down, only in the character of the advertisements which carry his favorite journal, so that he can buy it for the partial cost of a dollar a year.

Let us get out of the rut and into the march of progress, to our mutual benefit and advancement, even in the advertising line.

